# Quercetin Beneficial Role in the Homeostatic Variation of Certain Trace Elements in Dyslipidemic Mice

**DOI:** 10.1155/2022/3299505

**Published:** 2022-03-11

**Authors:** Florin Muselin, Romeo T. Cristina, Eugenia Dumitrescu, Alexandru O. Doma, Isidora Radulov, Adina A. Berbecea, Adina Horablaga, Florica E. Morariu, Dan N. Manea, Nicolae M. Horablaga

**Affiliations:** ^1^BUASMV “King Michael I of Romania”, Faculty of Veterinary Medicine, Department of Toxicology, Calea Aradului, No. 119, Timisoara 300645, Romania; ^2^BUASMV “King Michael I of Romania”, Faculty of Veterinary Medicine, Depts. of Pharmacology and Pharmacy, Calea Aradului, No. 119, Timisoara 300645, Romania; ^3^BUASMV “King Michael I of Romania”, Faculty of Agriculture, Department of Chemistry, Calea Aradului, No. 119, Timisoara 300645, Romania; ^4^BUASMV “King Michael I of Romania”, Faculty of Bioengineering of Animal Resources, Department of Ecology and Environmental Protection, Calea Aradului, No. 119, Timisoara 300645, Romania; ^5^BUASMV “King Michael I of Romania”, Faculty of Agriculture, Department of Environmental Engineering, Calea Aradului, No. 119, Timisoara 300645, Romania

## Abstract

**Background:**

Quercetin's role in the homeostasis of certain trace elements in dyslipidemia induced in mice was assessed.

**Methods:**

Forty BALB/*c* mice were allocated into 4 groups as follows: control; HFD, received fat diet; HFD + *Q* group, received HFD +500 mg/L quercetin; and blank control (*Q*)—normal food + 500 mg/L quercetin in drinking water.

**Results:**

By analyzing the values of total proteins, albumins, cholesterol, triglycerides, HDL cholesterol, LDL cholesterol, and the levels of several trace elements in blood and organs, we perceived a statistically significant increase (^*∗∗*^, *p* < 0.01) of TP, ALB, TC, TGE, and LDL-c. A nonsignificant decrease was ascertained to HDL-c value in the HFD and quercetin groups (*p* > 0.05). In the HFD group, all analyzed elements in the kidney and spleen increased, also Cu, Li, Mg, Mn, Pb, and of B, Ba, Cr, Cd, Cu, Fe, Li, Mn, Ni, Pb, and Zn in the heart increased, but furthermore, Ag, B, Ba, Cd, Cr, Fe, Ni, and Zn in blood, Ag and Zn in the liver, and Cd in the spleen decreased. In the HFD group who received quercetin, elements (except B) were decreased in kidney and liver, also increased Ag, Ba, Cr, Fe, Li, Ni, Zn in blood, but similarly, Ag, B, Ba, Cd, Cu, Mn, and Pb declined in the spleen and heart.

**Conclusions:**

Results proved the quercetin beneficial role.

## 1. Introduction

Dyslipidemia is part of metabolic syndrome and represents mainly a disorder of lipoprotein metabolism characterized by abnormalities of the following markers: increased low-density lipoprotein cholesterol (LDL-c), triglycerides (TGE), total cholesterol (TC), and decreased high-density lipoprotein cholesterol (HDL-c) [1,2].

Dyslipidemia is one of the factors that can be modified to prevent the development of cardiovascular diseases, atherosclerosis, stroke, and type 2 diabetes [1,3–8].

The 3, 3′, 4′, 5, 7-pentahydroxyflavone, known as quercetin, is a flavonoid that could be found widely in a large variety of fruits and vegetables [9–11].

Research on human and animal subjects has shown that quercetin has beneficial effects in cardiovascular disease, nervous system disease, and liver and kidney disease [9,12], since it possesses anti-inflammatory, antihypercholesterol, antisclerotic, and antiobesity properties [13,14]. Studies indicated a link between the disorders included in the metabolic syndrome and the status of trace elements. These are basically focused on diabetes, in humans [15,16] and animals [17], obesity, in humans [18–20] and animals [21], and those who enclose dyslipidemia, delimited to only one or not many trace elements such as copper, magnesium, and selenium [2,22–24]. Regarding the interrelationship of quercetin, trace elements, and metabolic syndrome, studies are scarce and also quite limited to diabetes [10] and obesity [21]. Consequently, the aim of this study (part of a larger one on this topic) was to find whether dietary quercetin supplementation plays a role in the homeostasis of trace elements in mice with induced dyslipidemia.

## 2. Methods

### 2.1. Animals and Experimental Protocol

Forty healthy BALB/*c* mice aging 2 months and with a mean weight of 25 ± 3 g obtained from the authorized biobase of the University of Medicine and Pharmacy “Victor Babes” Timisoara, Romania, were housed in standard polycarbonate cages (*l* × w × *h* = 750 mm × 720 mm × 360 mm). The environmental conditions were maintained at 22 ± 2°C, relative humidity of 55 ± 10%, and 12 h light/dark cycle. Before the start of the experiment, animals were kept in the same cages one week for acclimatization and were handled in accordance with directive 2010/63/EU on the handling of animals used for scientific purposes [25] and guidelines of the National Research Council (NRC) [26]. The experiment was approved by the Ethics Committee of the Faculty of Veterinary Medicine from Banat University of Agricultural Science and Veterinary Medicine from Timisoara (no.136/2021).

The mice were randomly distributed in four experimental groups (*n* = 10) as follows: • Group C: control, received normal food and distilled water; • group HFD: received high-fat diet (HFD) and distilled water; • group HFD + *Q*: received HFD and 500 mg/L quercetin in drinking water, and • group *Q*: as blank, received normal diet and 500 mg/L quercetin in drinking water.

The food and drinking water were provided ad libitum. The fat content of normal food was 5%, and HFD food lipid content was 45%. The quercetin (Quercetin 500 mg, Solaray, USA) was dissolved in 10 ml distilled water and ethanol in 4 : 1 ratio for 10 minutes, and then, distilled water was added until 1 litter; the final concentration was 500 mg × L-1. The quercetin solution was daily prepared to avoid precipitation.

In Table 1 the normal and high-fat diet content administered in this study are presented.

At the end of the experiment, all mice were euthanized by overdosing anesthetic agents using 300 mg × kg bw^−1^ of ketamine (Ketamine 10%, CP-Pharma, Burgdorf, Germany) and 30 mg × kg bw^−1^ of xylazine (Narcoxyl, Intervet International, Boxmeer, the Netherlands), in accordance with abovementioned directive [25], and the AEC SOP.26 Guidelines for Euthanasia of Mice and Rats [27], and blood and organs were collected. Blood samples were collected into clot activator BD Vacutainer (Ref no. 368975) and centrifuged for 10 min at 3000 × g to separate serum, following the methodology of Muselin et al. [28].

The measured parameters were the serum concentration of total protein (TP) and albumin (ALB), and markers of dyslipidemia: triglycerides (TGE), cholesterol (COL), HDL cholesterol (HDL-*c*), and LDL cholesterol (LDL-*c*).

The levels of silver (Ag), boron (B), barium (Ba), cadmium (Cd), chromium (Cr), copper (Cu), iron (Fe), lithium (Li), magnesium (Mg), manganese (Mn), nickel (Ni), lead (Pb), and zinc (Zn) in blood, liver, kidney, spleen, and heart were also analyzed.

### 2.2. Samples Analysis

Albumin (ALB), total protein (TP), cholesterol (TC), HDL cholesterol (HDL-*c*), and triglyceride (TGE) levels were determined by spectrophotometry method (BS3000 Semi-Automatic Chemistry Analyzer, Sinnowa Medical Science & Technology Co., Ltd., China) according to appropriate known standardized procedures, using commercially available kits from Chema Diagnostica (Italy) (REF: albumin–BC0100CH, total protein–TP0100CH, cholesterol–CTF100CH, HDL cholesterol–CD0400CH, and triglycerides–TRF100CH).

The LDL cholesterol (LDL-*c*) was calculated according to Friedewald's formula [20] [LDL-*c* = TC - HDL-*c* – (TG/5)].

For the determination of selected elements (Ag, B, Ba, Cd, Cr, Cu, Fe, Li, Mg, Mn, Ni, Pb, and Zn) samples, preparation was performed by microwave digestion.

The samples (0.2–0.5 g) were deposited in the digestion tubes adding 10 mL of concentrated nitric acid and 2 mL of hydrogen peroxide. The flasks were covered with a lid, inserted into the protective sleeve, and submitted to microwave digestion system (Multiwave GO, Anton Paar, GmbH, Austria), and the working schedule was the following: 20 min, 120°C, and 800 W. After digestion, the samples were placed into 25 mL rated flasks and added deionized water up to the mark. The analysis of selected element levels in the studied samples was performed using inductively coupled plasma-mass spectrometry (ICP-MS) at PlasmaQuant MS Elite Quadrupole (Analytic Jena, Germany) equipped with an ASPQ 3300 autosampler (Analytic Jena, Germany).

External calibration of the ICP-MS system was performed with 0.5, 1, 5, 25, 50, and 100 *µ*g × L^−1^ standards and prepared from ICP multielement standard solution IV (Merk Certipur, Germany).

Internal standardization was performed using 1 *µ*g × L^−1^ solutions of Sc - 45, Y – 89, and Re -185, prepared from Sc - 45, Y – 89, and Re -185 standard solution (Merk, Germany). The ICP-MS operating conditions were as follows: plasma flow—9.0 L/min, auxiliary flow—1.50 *L* × min^−1^, nebulizer flow—1.05 *L*× min^−1^, and sample uptake delay—60 sec.

### 2.3. Statistical Analysis

The obtained results were expressed as mean ± SEM (standard error of the means) and were analyzed by one-way ANOVA with the *Bonferroni's* multiple comparison test considering that the differences are statistically provided when *p* < 0.05 or lower, using the *GraphPad Prism 6.0* software (GraphPad Software, San Diego, USA).

## 3. Results

### 3.1. Serum Proteins

**Figure 1:**
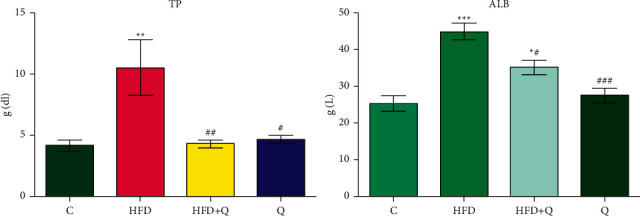
Levels of total proteins (TP) and albumin (ALB) in dyslipidemic mice and quercetin. Comparative to C group: ^*∗*^, *p* < 0.05, ^*∗∗*^, *p* < 0.01, and ^*∗∗∗*^, *p* < 0.001; comparative to HFD group: #*p* < 0.05 and ##*p* < 0.01.

TP and ALB (Figure 1) significantly increased (*p* < 0.001) in HFD compared to control group (TP: +154.90%, ALB: +77.78%) and significantly decreased in group that received HFD and quercetin (TP: -59.13%, *p* < 0.01; ALB: -21.85%, *p* < 0.05) but remaining to a level higher than in control, not significant (*p* > 0.05) for TP (+4.16%), and significant for ALB (+38.93%).

There were no significant (*p* > 0.05) differences between the blank quercetin (*Q*) group and the control group (TP: +12.39%, ALB: +8.51%).

### 3.2. Lipid Profile

**Figure 2:**
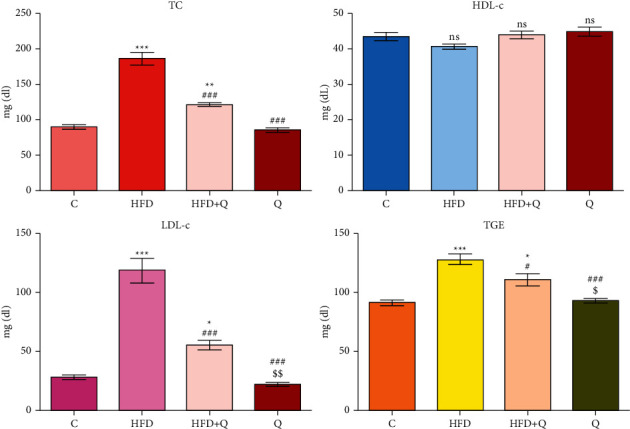
Dyslipidemic markers in mice receiving HFD and quercetin. Comparative to (C) ns-not significant; ^*∗*^, *p* < 0.05, ^*∗∗*^, *p* < 0.01, and ^*∗∗∗*^, *p* < 0.001. Comparative to HFD: #*p* < 0.05 and ###*p* < 0.01. Comparative to HFD + *Q*: $ *p* < 0.055 and $$ *p* < 0.01.

The lipid profile is presented in Figure 2. Total cholesterol (TC) significantly (*p* < 0.001) increased in HFD mice compared to controls (+108.40%) and significantly decreased (*p* < 0.001) when quercetin was administered to mice (-34.86%). Even if the decrease was highly significant, the level still remains significantly (*p* < 0.01) higher in this group in comparison with the control (+35.74%).

The level of TC was lower in blank *Q* group compared to the control (-4.69%), but the differences between them were not significant (*p* > 0.05). The same dynamic was noted regarding the LDL-*c* and TGE, namely, a significant increase in LDL-*c* and TGE in groups receiving HFD compared to control (LDL-*c*: +331.05%, *p* < 0.001; TGE: +41.67, *p* < 0.01) and significant (*p* < 0.05) decrease in LDL-*c* and TGE when the quercetin was added compared to HFD group (LDL-*c*: -53.63%, TGE: -13.59%), but the levels remains significantly higher for both lipids compared to control group (LDL-*c*: +99.85%, *p* < 0.001; TGE: +22.41%, *p* < 0.05).

There were observed mild differences between the *Q* group and the control, lower LDL-*c* (-21.53%), and higher TGE (+2.67%) in the *Q* group compared to the control group, the differences being statistically not significant (*p* > 0.05). The HDL-*c* level decreased in HFD group (-6.32%) and increased in the group that received quercetin (+1.16%) compared to control, the increase being higher compared to the HFD group (+7.99%), and in all cases, the differences were not significant (*p* > 0.05). A nonsignificant increase (*p* > 0.05) was recorded in the *Q* group compared to the control (+3.17%).

### 3.3. Trace Element Levels in Blood and Organs

The analyzed trace element levels in different experimental groups are presented in Table 2.

#### 3.3.1. Blood

In the group that received HFD was reported, compared to control, a significant decrease in Ag (−53.82%, *p* < 0.05), B −86.23%, *p* < 0.01), Ba (−94.39%, *p* < 0.001), Cd (−61.81%, *p* < 0.001), and Fe (−58.55%, *p* < 0.001), nonsignificant (*p* > 0.05) decrease in Cr (−11.54%), Ni (−36.46%), and Zn (−16.73%), and the nonsignificant (*p* > 0.05) increase in Cu (+92.49%), Li (+8,14%), Mn (+102.31%), and Pb (+35.22%), but significant (*p* < 0.01) increase in Mg (+120.85%). In HFD group that received quercetin, comparative to HFD group, we observed nonsignificant (*p* > 0.05) increase in Ag (+7.24%), Ba (+14.48%), Cr (+18.14%), Fe (+51.21%), Li (+22.77%), Ni (+24.11%), and Zn (+22.09%), nonsignificant (*p* > 0.05) decrease in B (−52.90%), Cd (−16.66%), Cu (−5.26%), Mn (−29.95%), and Pb (−33.12%), and significant (*p* < 0.01) decrease in Mg (−45.99%). Even if it detected a tendency to re−establish the homeostasis of these trace elements, the levels did not reach that of the control group level, remaining different compared to control group (HFD + *Q*/C), significant for Ag (−50.47%, *p* < 0.05), B (−93.51%, *p* < 0.001), Ba (−93.41%, *p* < 0.001), Cd (−99.65%, *p* < 0.001), and Fe (−37.32%, *p* < 0.05) and not significant (*p* > 0.05) for Cr (+4.50%), Cu (+82.36%), Li (+32.77%), Mg (+20.81%), Mn (+41.72%), Ni (−21.14%), Pb (−9,56%), and Zn (+1.65%).

There also have been ascertained some differences between control and blank quercetin group, namely, the decrease in Ag (-41.96%, *p* > 0.05), Ba (-36.15%, *p* < 0.05), Cd (-82.72%, *p* < 0.001), Pb (-37.24%, *p* > 0.05), and Zn (-56.44%, *p* < 0.01), respectively, and increase in B (+4.21%, *p* > 0.05), Cr (+136%, *p* > 0.05), Cu (+25.99%, *p* > 0.05), Li (+33.21%, *p* > 0.05), Mg (+132.06%, *p* > 0.05), Mn (+210.53%, *p* < 0.001), and Ni (+16.97%, *p* > 0.05).

#### 3.3.2. Liver

In the liver, the levels of almost all analyzed elements were increased in HFD group compared to control, not significant (*p* > 0.05) for B (+46.57%), Ba (+17.98%), Cr (+59.45%), Cu (+23.58%), Li (+20.18%), Mg (+12.01%), Mn (+13.23%), and Ni (+21.15%), and significant for Fe (+66.76%, *p* < 0.001) and Pb (+40.87%, *p* < 0.05).

The exception was for Ag and Zn, which present a decrease (Ag: -23.70%, *p* > 0.05; Zn: -86.70%, *p* < 0.001), and Cd, which remain at the same level as in control.

In the group fed with HFD and received quercetin compared to the group only fed with HFD, we observed the decrease in analyzed elements, the exception being B and Zn, which were not significantly (*p* > 0.05) increased (B: +3.94%, Zn: +4.25%). For the remainder elements, the decrease was not significant (*p* > 0.05) for Ag (-10.23%), Cd (-40.90%), Cr (-15.92%), and Fe (-16.38%), Zn (-17.55%), significant (*p* < 0.05) for Ba (-39.03%), Cu (-40.56%), Li (-28.14%), and Mn (-44.11%), and highly significant for Mg (-32.49%, *p* < 0.01), Ni (-41.79%, *p* < 0.001), and Pb (-39.58%, *p* < 0.001). Even if overall, there were no significant differences between the blank quercetin group and the control, and we still detected significantly higher levels (*p* < 0.001) of B (+109.78%) and Ba (+77.60%) and significantly lower levels of Ni (-37.14%, *p* < 0.01) and Pb (-86.69%, *p* < 0.001) in mice from blank quercetin group compared to control group.

#### 3.3.3. Kidney

In kidneys, it an increase was observed in the analyzed elements in HFD group compared to control, not significant (*p* > 0.05) for Ag (+39.19%), Cd (+76.92%), Cr (+53.99%), Cu (+54.92%), and Mg (+25.31%), Zn (+54.4%), and significant for Ba (+63.32%, *p* < 0.05), Fe (+71.32%, *p* < 0.001), Li (+59.88%, *p* < 0.001), Mn (+32.88%, *p* < 0.01), Ni (+48.67%, *p* < 0.001), and Pb (+97.43%, *p* < 0.01). When quercetin was administered in HFD group, we recorded the decrease in studied trace elements, not significant (*p* > 0.05) for Ag (-13.16%), B (-17.40%), Ba (-24.34%), Cd (-42.99%), Cu (-15.37%), Fe (-15.58%), Mg (-24.91%), Ni (-9.57%), Pb (-35.21%), and Zn (-4.21%), but significant for Li (-19.92%, *p* < 0.05) and Mn (-26.79%, *p* < 0.01). Nonsignificant (*p* > 0.05) differences were noted between the two control groups, with the exception of Cr, which was significantly (*p* < 0.001) increased in blank quercetin group compared to control (+215.06%).

#### 3.3.4. Spleen

In the spleen of HFD mice, we recorded a decrease in the analyzed elements compared to control with a slight and nonsignificant (*p* > 0.05) increase in Cd (+1.82%). Even the decrease was evident, it was not statistically significant (*p* > 0.05) for Ag (-41.22%), B (-29.12%), Cr (-43.72%), Cu (-16.14%), Mg (-29.84), Pb (-6.52%), and Zn (-35.72%), but the decrease was significant for Ba (-54.22%, *p* < 0.001), Fe (-44.01%, *p* < 0.05), Li (-58.15%, *p* < 0.01), Mn (-27.68%), and Ni (-45.54%, *p* < 0.01).

The administration of quercetin in HFD group was followed by changes in the distribution of elements in spleen, namely, the continuous decrease compared to HFD group, not significant (*p* > 0.05) for Ag (-1.30%), Ba (-50.35%), Cu (-16.35%), and Mn (-30.92%), but significant for Cd (-54.71%, *p* < 0.01) and Pb (-64.04%, *p* < 0.001). In the same group, we also noted a not significant (*p* > 0.05) increase in B (+34.90%), Cr (+20.28%), Fe (+11.40%), Mg (+35.22%), Ni (+47.26%), and Zn (+31.14%), and significant (*p* < 0.001) increase in Li (+439.77%).

Regarding the differences between control and blank quercetin group, some mild differences were also observed in blank quercetin group as follows: nonsignificant (*p* > 0.05) decrease in Ag (-13.53%), Ba (-19.69%), Cd (-13.24%), and Ni (-24.87%), significant decrease in Fe (-43.21%, *p* < 0.05), Mn (-66.26%, *p* < 0.001), and Pb (-87.13%, *p* < 0.001), but, on the other hand, we recorded a nonsignificant (*p* > 0.05) increase in Cu (+1.24%) and a significant increase in B (+162.03%, *p* < 0.001), Cr (+155.50%, *p* < 0.001), Li (+169.78%, *p* < 0.001), Mg (+59.81%, *p* < 0.05), and Zn (+158.62%, *p* < 0.001).

#### 3.3.5. Heart

In the group that received HFD, compared to the control, there were observed a nonsignificant modification of analyzed trace elements in the heart and a significant increase in toxic trace elements Cd (+109.91%, *p* < 0.05) and Pb (+49.11%, *p* < 0.01). For the remainder of trace elements, we noted a decrease in Ag (-1.07%) and Mg (-23.12%) and an increase in B (+34.64%), Ba (+10.8%), Cr (+16.91%), Cu (+27.91%), Fe (+28.68%), Li (+17.35%), Mn (+6.81%), Ni (+8.57%), and Zn (+26.37%).

When quercetin and HFD were administered, a nonsignificant (*p* > 0.05) increase in B (+15.78%), Fe (+3.29%), Li (+64.44%), Mg (+5.81%), and Zn (+4.35%) was observed and also a nonsignificant (*p* > 0.05) decrease in Ag (-1.06%), Ba (-23.4%), Cd (-30.31%), Cr (-7.15%), Cu (-7.71%), Mn (-16.67%), Ni (-22.45%), and Pb (-20.83%) compared with mice from HFD group without quercetin was observed.

There were also recorded differences in blank quercetin group compared to the normal control group as follows: highly significant increase in Ag (+53.55%, *p* < 0.01), B (+193.67%, *p* < 0.05), Cr (+232.92%, *p* < 0.05), Cu (+145.56%, *p* < 0.01), Li (+241.76%, *p* < 0.001), Mg (+243.88%, *p* < 0.001), and Mn (+159.47%, *p* < 0.05), Zn (+71.73%, *p* < 0.01), and nonsignificant (*p* > 0.05) increase in Ba (+45.84%), and, on the other side, nonsignificant (*p* > 0.05) decrease in Cd (-23.96%) and Ni (-4.87%), but a significant (*p* < 0.01) decrease in Fe (-47.12%) and Pb (-49.28%).

## 4. Discussion

Proteins are the most abundant components of blood serum or plasma, having many essential physiological functions [29].

Among them, albumins, the most abundant serum proteins, act as carriers of hormones, vitamins, lipids, and minerals in the circulatory system and are involved in the regulation of cellular activity and immune system [30,31]. In this study, we observed a significant increase in TP and ALB in the HFD group compared to the control and a significant decrease when quercetin was administered. The increase was directly correlated with the levels of TC, TGE, and LDL-*c* and indirectly correlated with HDL-*c* level.

Metabolic disorders are typically associated with insulin resistance, and because insulin regulates protein dynamics, dyslipidemia would alter protein synthesis [32], being demonstrated that the syntheses of total protein and serum albumins are sensitive to nutritional status [32] as was also observed in our study.

In our study, we noted a significant increase in TC, LDL-*c,* and TGE and a decrease in HDL-*c* in groups that received a fat diet compared with the control and blank group, being in accordance with the studies of other researchers [2,4,18,22].

Lipid energy is transported in the blood in different forms, including free fatty acid (FFA). FFA represents the main lipid fuel in the body, and by increasing their concentration, it will cause insulin resistance, endothelial dysfunction, an increase in the production of very low-density lipoprotein, and the development of dyslipidemia [33].

FFA has been shown to produce a defect in insulin-stimulated glucose transport and/or phosphorylation, which is caused by a defect in insulin signaling [34].

Plasma FFA can easily pass to cells where they could be oxidized to generate energy in the form of ATP or re-esterified to be stored as triglycerides, this being a possible explanation of the increase in TGE in the HFD group [31,34].The increase in serum TC might be a consequence of the mobilization of free fatty acids from the adipose tissue to the bloodstream, increasing the level of acetyl CoA, and this increases the synthesis of cholesterol [35].

We observed a significant (*p* < 0.01) decrease in TC, LDL-*c,* and TGE followed by a nonsignificant (*p* > 0.05) increase in HDL-*c* in groups that received quercetin compared to control.

Mbikay et al. [36] attributed the cholesterol-lowering effect of quercetin to the fact that this flavonoid augments hepatic LDL receptors and protein convertase subtilisin/kexin type 9 (PCSK9), which could be a possible explanation of the cholesterol decrease in our study.

Some macroelements such as Mg and Fe and certain trace elements such as B, Cr, Cu, Zn [37], and Cd [38,39] enhance insulin action by activating insulin receptor sites, thus playing a specific role in the pathogenesis of diabetes [33,40] and dyslipidemia [33]. In our study, we observed in dyslipidemic mice an increase *(even if some of them are not statistically significant)* in B, Cd, Cr, Cu, and Fe in kidney, liver, and heart, in Mg in blood, kidney, and liver, and in Zn in kidney and heart levels that were alleviated by the administration of quercetin.

Zhu et al. [41] did not observe a significant correlation between dyslipidemia and the levels of Cr, Mn, Pb, Fe, Cu, and Zn in the hair of elders from China, but, in our study, these trace elements were higher in the liver, kidney, and heart of dyslipidemic mice showing a positive correlation between dyslipidemia and these trace elements' accumulation in aforementioned organs. A decrease in levels was observed for these trace elements when quercetin was administered, the exception being for Fe and Zn in the heart, which was remained not significantly higher than those from dyslipidemic mice.

Tinkov et al. [20] observed that obesity is associated with the lower levels of Fe, Mg, and Zn and a higher level of Cu in serum, hair, and urine and showed a direct correlation of these levels with the lipid profile of obese subjects. Cu and Zn are essential components of the functional groups of several enzymes that may play a key role in the prevention of atherosclerosis [22].

Lopes et al. [42] recorded a nonsignificant increase in serum Cu and Zn in hyperlipidemic people from Lisbon compared to normolipidemic ones, partially similar to our findings, in which we observed a nonsignificant increase in Cu and a nonsignificant decrease in Zn in the blood of dyslipidemic mice, being in accordance with the findings of Abiaka et al. [2], which have found a positive link between hypercupremia and hyperlipidemia in humans.

Tamrakar et al. [15] observed a significant increase in Cu and a decrease in Mg in diabetic dyslipidemic patients pointing out that these findings are related to oxidative stress.

In our study, we recorded a not significant increase in Li in blood, liver, and heart, and Ni in liver and heart, and also a significant increase in both in the kidney of dyslipidemic mice compared to control levels that were changed in dyslipidemic mice that received quercetin.

There are studies that pointed out that high levels of lithium [43] and nickel [44] in blood after administration of these elements are incriminated to produce dyslipidemia. Furthermore, administration of some flavonoids (naringin) reduced the accumulation of Ni in blood and liver [44] being partial in accordance with our findings related to the levels of Ni in blood and liver of dyslipidemic mice and the effects of quercetin.

Also, Shumakova et al. [21] observed a positive correlation between lipid profile and the accumulation of Cr in the liver and Ni in the kidney of obese rats, similar to our findings, and a negative correlation with the accumulation of Fe, Zn, and Mg in the liver, and Mg, Cu, and Zn in the kidney of obese rats, being partial in accordance with our findings in dyslipidemic mice. We did not find studies related to the Ag and Ba levels in metabolic syndrome disorders, but we observed that Ag and Ba levels are significantly decreased in the blood, increased in kidneys, and not significantly decreased in spleen and heart of dyslipidemic mice, and were increased in the blood and more decreased in the studied organs when quercetin was administered.

Excessive intake of flavonoids may cause a decrease in essential trace elements and their related enzyme activities, being observed that flavonoids as transition metal chelators may cause a decrease in trace minerals, such as Fe, Cu, Zn [42], and Ni [21].

In our study, we recorded in the HFD group that also received quercetin a decrease in all analyzed elements in the liver and kidney and decrease in B, Cd, Cu, Mg, Mn, and Pb in the blood, Ag, Ba, Cd, Cu, Mn, and Pb in the spleen, and Ag, Ba, Cd, Cr, Cu, Mn, Ni, and Pb in the heart. We also observed a very important and significant decrease in the toxic trace elements Cd and Pb in blood and organs of HFD mice that received quercetin. Furthermore, we observed an increase in Ag, Ba, Cr, Fe, Ni, and Zn in the blood, Li and Ni in the liver, Fe, Li, Mg, Ni, and Zn in the spleen, and Fe, Li, Mg, and Zn in the heart of HFD mice when supplemented with quercetin. The imbalance between the concentrations of different trace elements, which were observed in different organs, could be explained by a selective influence of the hyperglycemic-hyperinsulinemic state on the ability of different organs to extract these metals [10], being demonstrated that flavonoids have an insulin-mimic property [45].

Jaccob and Hussain [46] recorded a significant increase in Fe, Cu, and Zn in the brain, liver, and kidney of rats that received quercetin compared to the control group, and Krol et al. [47] recorded a decrease in Cu, Fe, and Zn in the liver, kidney, and spleen of diabetic rats that received mulberry leaf extract containing quercetin, which are only partial in accordance with our findings.

Flavonoids can bind metal ions in vivo, thus reducing their uptake and storage in internal organs [43] as was observed in our study. The flavonoid-metal interaction, which can affect the metallothionein level, could have a profound impact on the trace element homeostasis in the organism [48].

All these modifications of studied elements in the blood and organs of dyslipidemic mice indicate the very complex nature of the many processes that underline the homeostasis of macro and trace elements, which take place at the level of ion competition for binding sites and transport systems [21].

## 5. Conclusions

Quercetin dietary supplemented to high-fat diet mice (HFD) was followed by a significant decrease in TC, LDL-*c,* and TGE, decrease in all analyzed elements in the liver and kidney, and decrease in B, Cd, Cu, Mg, Mn, and Pb in the blood, Ag, Ba, Cd, Cu, Mn, and Pb in the spleen, and Ag, Ba, Cd, Cr, Cu, Mn, Ni, and Pb in the heart. On the other hand, an increase in Ag, Ba, Cr, Fe, Ni, and Zn in the blood, Li and Ni in the liver, Fe, Li, Mg, Ni, and Zn in the spleen, and Fe, Li, Mg, and Zn in the heart of HFD mice when was supplemented with quercetin was reported.

The most perceptible was the significant decrease in the toxic trace elements Cd and Pb in blood and organs of HFD mice that received quercetin, permitting us to conclude that the quercetin plays a beneficial role in lipid profile regulation and homeostasis of studied macro and trace elements in mice with induced dyslipidemia.

## Figures and Tables

**Table 1:** The content of normal and high-fat diet given to animals in this study.

Content	Normal diet	High-fat diet
Protein	18 g%	18 g%
Carbohydrate	75 g%	35 g%
Fat	5 g%	45 g%
Minerals		
Calcium	3.8 g/kg	3.6 g/kg
Chloride	0.7 g/kg	0.7 g/kg
Copper	3.2 mg/kg	3.1 mg/kg
Iron	39.5 mg/kg	40.1 mg/kg
Magnesium	0.39 g/kg	0.4 g/kg
Manganese	45.2 mg/kg	45.1 mg/kg
Potassium	4.8 g/kg	4.7 g/kg
Selenium	0.19 mg/kg	0.18 mg/kg
Sodium	525 mg/kg	522 mg/kg
Zinc	22.6 mg/kg	21.2 mg/kg

**Table 2:** Levels of macro and trace elements in blood, liver, kidney, spleen, and heart of dyslipidemic mice treated with quercetin.

Element	Organ	Group (*X*±SEM)			
		C	HFD	HFD + *Q*	Q
**Ag** (*µ*g × g^−1^)	Blood	672.2 ± 60.82	310.4 ± 53.86^*∗*^	332.9 ± 82.06^*∗*^	390.1 ± 55.56
	Kidney	272.2 ± 11.39	378.9 ± 7.96	329.0 ± 31.06	1002 ± 120.9^###,$$$^
	Liver	216.4 ± 38.11	165.1 ± 16.15	148.2 ± 24.72	105.2 ± 2.78^*∗*^
	Spleen	680.4 ± 150.4	399.9 ± 31.77	394.7 ± 102.7	588.3 ± 66.6
	Heart	416.8 ± 5.65	412.3 ± 8.92	408.4 ± 24.91	640.0 ± 68.98^*∗∗*^^,##,$$^

**B** (*µ*g × kg^−1^)	Blood	4419 ± 855	608.8 ± 142.3^*∗∗*^	286.7 ± 55.86^*∗∗∗*^	4605 ± 583.8^###,$$$^
	Kidney	324.8 ± 15.47	612.5 ± 51.28^*∗∗∗*^	505.9 ± 43.51^*∗*^	290.1 ± 30.09^$$^
	Liver	187.0 ± 15.64	274.1 ± 5.95	284.9 ± 32.8	392.3 ± 31.15^*∗∗∗*^^,#,$^
	Spleen	880.8 ± 103.8	624.3 ± 70.76	842.2 ± 104.4	2308 ± 175.3^*∗∗∗*^^,###,$$$^
	Heart	507.7 ± 37.55	683.6 ± 56.29	791.5 ± 60.35	1491 ± 293.1^*∗*^^,#,$^

**Ba** (*µ*g × g^−1^)	Blood	693.32 ± 74.4	39.89 ± 10.54^*∗∗∗*^	45.67 ± 9.93^*∗∗∗*^	442.64 ± 72.73^*∗*^^,###,$$^
	Kidney	5.18 ± 0.12	8.46 ± 1.03^*∗*^	6.40 ± 0.69	4.31 ± 0.15^##^
	Liver	3.17 ± 0.41	3.74 ± 0.22	2.28 ± 0.13^#^	5.63 ± 0.37^*∗∗∗*^^,##,$$$^
	Spleen	15.49 ± 1.78	7.09 ± 0.78^*∗∗∗*^	3.52 ± 0.15^*∗∗∗*^	12.52 ± 0.87^#,$$$^
	Heart	7.94 ± 0.33	8.80 ± 0.41	6.74 ± 0.23	11.58 ± 1.58^$$^

**Cd** (*µ*g × g^−1^)	Blood	1.10 ± 0.06	0.42 ± 0.06^*∗∗∗*^	0.35 ± 0.05^*∗∗∗*^	0.19 ± 0.02^*∗∗∗*^
	Kidney	1.17 ± 0.2	2.07 ± 0.35	1.18 ± 0.16	0.41 ± 0.04^##^
	Liver	1.11 ± 0.26	1.11 ± 0.08	0.65 ± 0.11	0.49 ± 0.1
	Spleen	2.19 ± 0.43	2.23 ± 0.19	1.01 ± 0.16^*∗*^^,#^	1.90 ± 0.08
	Heart	1.21 ± 0.22	2.54 ± 0.4^*∗*^	1.77 ± 0.11	0.92 ± 0.11^##^

**Cr** (*µ*g × g^−1^)	Blood	153.3 ± 29.13	135.6 ± 21.9	160.2 ± 19.59	361.8 ± 84.68^#^
	Kidney	111.5 ± 5.66	171.7 ± 12.36	137.6 ± 5.78	483.0 ± 102.6^*∗∗∗*^^,$$^
	Liver	44.25 ± 7.35	70.56 ± 6.62^*∗*^	59.32 ± 3.21	58.32 ± 3.09
	Spleen	283.4 ± 39.54	160.2 ± 14.26	192.7 ± 38.83	724.1 ± 61.3^*∗∗∗*^^,###,$$$^
	Heart	164.9 ± 3.52	192.8 ± 16.14	179.0 ± 9.98	549.0 ± 165.5^*∗*^

**Cu** (*µ*g × kg^−1^)	Blood	700.8 ± 102.7	1349 ± 139.1	1278 ± 283.3	883.0 ± 186.9
	Kidney	1096 ± 63.55	1698 ± 106.6	1437 ± 58.6	2670 ± 263.1^$$$^
	Liver	650.5 ± 54.72	803.9 ± 41.96	477.8 ± 35.02^#^	854.8 ± 116.0^$$^
	Spleen	1932 ± 87.23	1620 ± 141.1	1355 ± 202.2	1956 ± 44.94^$^
	Heart	1580 ± 53.98	2021 ± 146.3	1865 ± 122.6	3880 ± 667.5^*∗∗*^^,#,$$^

**Fe** (mg × g^−1^)	Blood	101.04 ± 12.2	41.88 ± 6.3^*∗∗∗*^	63.33 ± 6.26^*∗*^	73.14 ± 1.39
	Kidney	111.6 ± 6.46	191.2 ± 14.2^*∗∗∗*^	161.4 ± 6.35^*∗∗*^	62.9 ± 3.35^$$$^
	Liver	49.91 ± 5.51	83.23 ± 3.89^*∗∗∗*^	69.59 ± 3.93^*∗*^	34.29 ± 2.06^###,$$$^
	Spleen	328.22 ± 27.45	183.79 ± 19.5^*∗*^	204.75 ± 50.45	186.39 ± 10.08^*∗*^
	Heart	164.43 ± 9.45	211.6 ± 17.85	218.57 ± 9.21^*∗*^	86.95 ± 9.26^*∗∗*^^,###,$$$^

**Li** (*µ*g × kg^−1^)	Blood	2014 ± 472.8	2178 ± 238.7	2674 ± 302.7	2683 ± 146.3
	Kidney	570.3 ± 41.99	911.8 ± 58.94^*∗∗∗*^	730.1 ± 35.96^#^	506.0 ± 26.46^$^
	Liver	343.8 ± 22.86	413.2 ± 22.91	296.9 ± 24.73^#^	251.1 ± 20.44^###^
	Spleen	2115 ± 74.49	885.0 ± 73.23^*∗∗*^	4777 ± 306.5^*∗∗∗*^^,###^	5706 ± 267.0^*∗∗∗*^^,###^
	Heart	893.9 ± 27.08	1049 ± 78.14	1725 ± 333.5	3055 ± 177.1^*∗∗∗*^^,###,$$^

**Mg** (*µ*g × kg^−1^)	Blood	20.71 ± 1.48	45.74 ± 3.39^*∗∗*^	25.02 ± 5.31^##^	48.06 ± 2.77^$$^
	Kidney	30.30 ± 2.24	37.97 ± 2.54	28.51 ± 0.56	43.51 ± 6.02^$^
	Liver	15.58 ± 0.86	17.45 ± 1.33	11.78 ± 0.51^*∗*^^,##^	12.56 ± 0.62^##^
	Spleen	47.31 ± 2.26	33.19 ± 2.87	44.88 ± 7.26	75.61 ± 6.58^*∗*^^,###,$$^
	Heart	51.02 ± 4.72	39.22 ± 4.04	41.5 ± 3.23	175.45 ± 20.79^*∗∗∗*^^,###,$$^

**Mn** (*µ*g × kg^−1^)	Blood	79.38 ± 8.88	160.6 ± 27.45	112.5 ± 18.44	246.5 ± 19.14^*∗∗∗*^^,$$$^
	Kidney	53.21 ± 2.88	70.71 ± 4.75^*∗∗*^	51.76 ± 2.43^##^	46.85 ± 1.53
	Liver	32.57 ± 2.71	36.88 ± 2.17	20.61 ± 0.49^*∗*^^,#^	41.54 ± 3.06^$$$^
	Spleen	92.16 ± 6.03	66.65 ± 5.14^*∗*^	46.04 ± 5.36^*∗∗∗*^	31.09 ± 4.91^*∗∗∗*^^,#^
	Heart	74.38 ± 1.66	79.45 ± 5.82	66.2 ± 1.97	193.0 ± 42.9∗^,#,$$^

**Ni** (*µ*g × kg^−1^)	Blood	199.1 ± 15.77	126.5 ± 18.81	157.0 ± 19.09	232.9 ± 18.9^##^
	Kidney	283.7 ± 14.27	421.8 ± 31.98^*∗∗∗*^	381.4 ± 9.15^*∗*^	452.2 ± 16.52
	Liver	166.9 ± 18.11	202.2 ± 10.06	117.7 ± 6.21^*∗*^^,###^	104.9 ± 6.96^*∗∗*^^,###^
	Spleen	790.2 ± 85.42	430.3 ± 36.87^*∗∗*^	633.7 ± 46.6	593.6 ± 54.76
	Heart	459.3 ± 19.95	498.7 ± 42.59	386.7 ± 16.08	436.9 ± 62.76

**Pb** (*µ*g × kg^−1^)	Blood	128.6 ± 11.55	173.9 ± 24.72	116.3 ± 25.95	80.70 ± 8.59^#^
	Kidney	47.49 ± 2.11	93.76 ± 10.75^*∗∗*^	60.74 ± 11.3	15.91 ± 1.49^###,$$^
	Liver	29.16 ± 2.88	41.08 ± 1.53^*∗*^	24.82 ± 3.07^###^	3.88 ± 0.82^*∗∗∗*^^,##,$$$^
	Spleen	121.9 ± 13.29	113.9 ± 12.16	40.95 ± 4.99^*∗∗∗*^^,###^	15.68 ± 3.73^*∗∗∗*^^,###^
	Heart	81.28 ± 7.30	121.1 ± 2.75^*∗∗*^	95.87 ± 9.67	41.22 ± 5.36^*∗∗*^^,###,$$$^

**Zn** (*µ*g × kg^−1^)	Blood	6872 ± 408	5722 ± 840.6	6986 ± 211.3	2993 ± 499.6^*∗∗*^^,#,$$^
	Kidney	815.4 ± 61.83	1259 ± 106.8	1206 ± 36.93	2402 ± 166.1^###,$$$^
	Liver	3875 ± 377.7	624.8 ± 34.04^*∗∗∗*^	515.1 ± 47.7^*∗∗∗*^	4040 ± 488.0^###,$$$^
	Spleen	2388 ± 137.6	1535 ± 45.57	2013 ± 113.6	6176 ± 505.8^*∗∗∗*^^,###,$$$^
	Heart	1164 ± 43.27	1471 ± 143.1	1535 ± 138.4	1999 ± 60.67^*∗∗*^^,#,$^

Comparative to C: ^*∗*^, *p* < 0.05, ^*∗∗*^*p* < 0.01, and ^*∗∗∗*^, *p* < 0.001. Comparative to HFD: #*p* < 0.05, ##*p* < 0.01, and ###*p* < 0.001. Comparative to HFD + *Q*: $ *p* < 0.05, $$ *p* < 0.01, and $$$ *p* < 0.001.

## Data Availability

All data are included in the attached manuscript.
